# Supplementary material for: Tardigrade small heat shock proteins can limit desiccation-induced protein aggregation
Source: Commun Biol. 2023 Jan 30;6:121. doi: 10.1038/s42003-023-04512-y (PMC9887055; doi:10.1038/s42003-023-04512-y)
Supplement: Supplementary file 2 — Supplementary Information [file 42003_2023_4512_MOESM2_ESM.pdf]

Supplementary Information

Figure S1

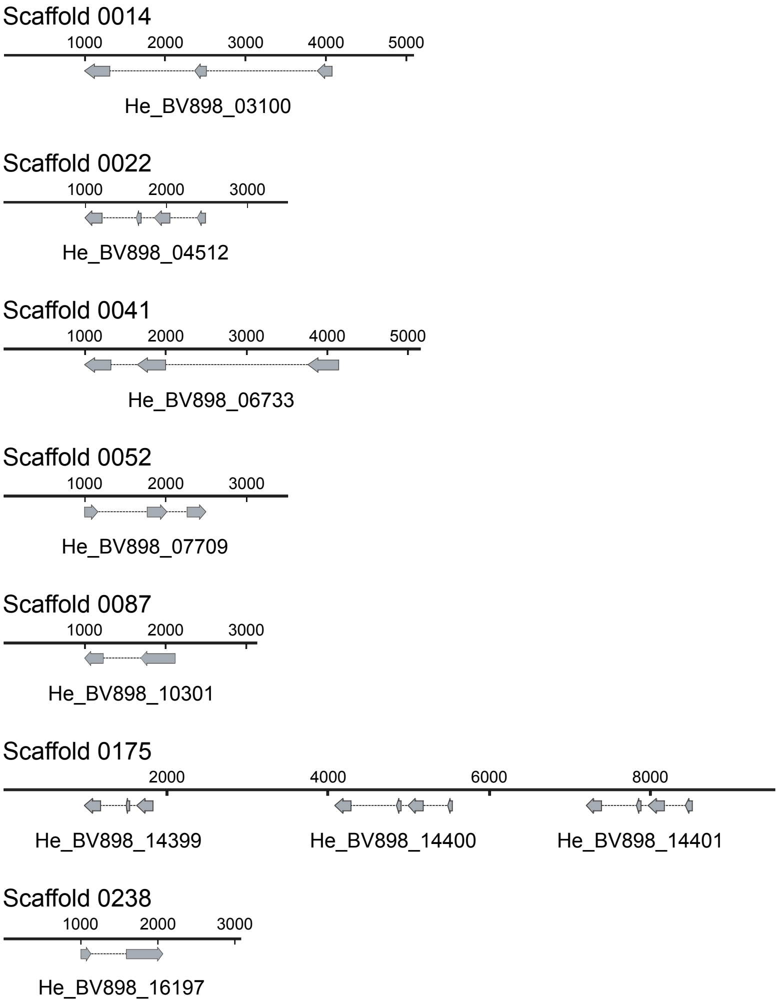

Figure S1. The exons of each sHSP are depicted on genomic scaffolds from Yoshida *et al.* 2017. Each gene is comprised of two to four exons. BV898_14399 (HSP17), BV898_14400 (HSP19), and BV898_14401 (HSP20) are located contiguously on the same scaffold.

Figure S2

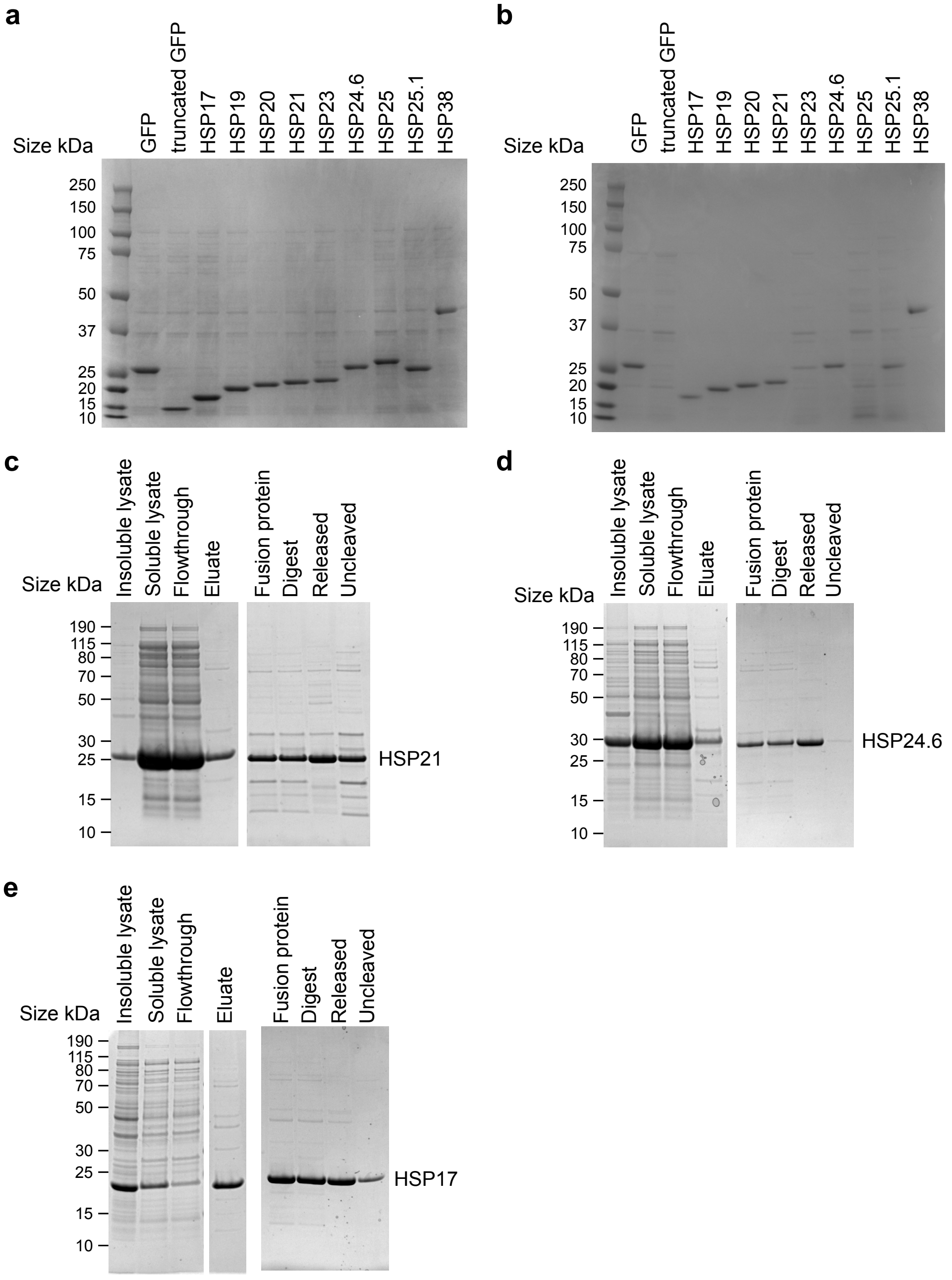

Figure S2. sHSP bacterial expression and purification of HSP17, HSP21, and HSP24.6. a) sHSPs and controls were expressed in BL21 *E. coli*. Total lysate was loaded for SDS-PAGE analysis. b) Soluble protein from bacterial lysates indicates limited solubility of truncated GFP, HSP23, and HSP25. c-e) Purification of HSP21 (c), HSP24.6 (d), and HSP17 (e). The first gel in each panel shows fractions following initial separation of soluble and insoluble lysate, the flowthrough from the column, and the bound protein that was eluted. The second gel shows the digested protein, protein released from the column, and uncleaved protein that was retained on the column due to presence of a His tag. The final protein is in the released sample.

Figure S3

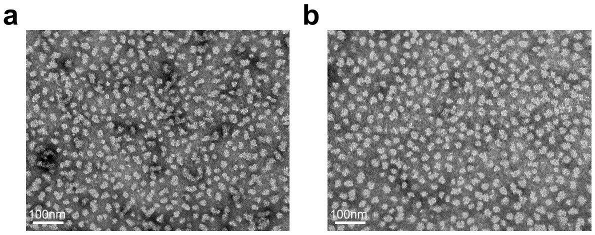

Figure S3. Formation of large complexes of HSP21 and HSP24.6 was not buffer-dependent. To ensure that oligomeric assemblies are not due to protein instability in water, we also imaged protein diluted in TEN buffer and saw similar assemblies of HSP21 (a) and HSP24.6 (b).

Figure S4

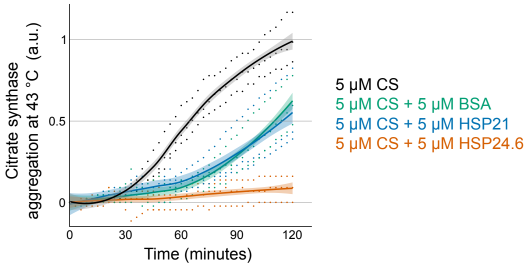

Figure S4. 5 µM HSP21, HSP24.6, and BSA limit heat-induced aggregation of citrate synthase at 43 °C. Aggregation is plotted as the change in absorbance at 340nm relative to that of 5 µM citrate synthase alone at 2 hr. Datapoints from three biological replicates and fitted lines with 95% confidence intervals are shown.

Figure S5

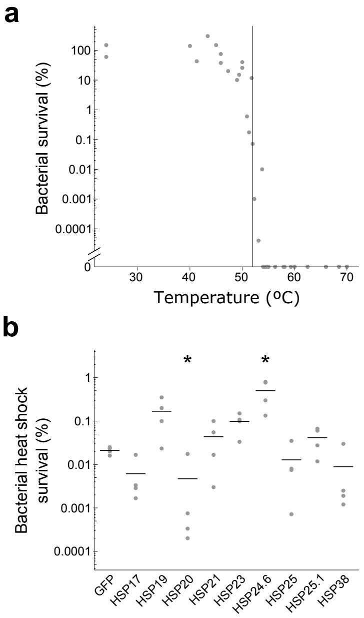

Figure S5. Heat shock survival of bacteria expressing sHSPs. a) Survival of GFP-expressing bacteria is plotted across a range of temperatures. b) Survival of bacteria expressing GFP or tardigrade sHSPs at 52 °C. Only HSP20 (p=0.01, Dunnett's test, n=4) and HSP24.6 (p=0.02, Dunnett's test, n=4) were significantly different from GFP-expressing controls. * p<0.05.

Figure S6

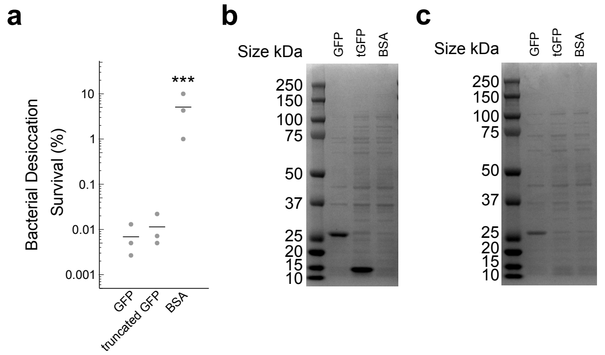

Figure S6. BSA improves bacterial desiccation survival. a) Desiccation survival of bacteria is plotted. Bacteria expressing BSA had a higher rate of survival than GFP-expressing controls (p<0.001, Dunnett's test, n=3). b) Total protein lysate from bacteria expressing GFP, truncated GFP, and BSA indicate strong expression of GFP and tGFP, but not BSA. c) Soluble protein from lysates in B include high levels of GFP, but not tGFP or BSA.

Figure S7

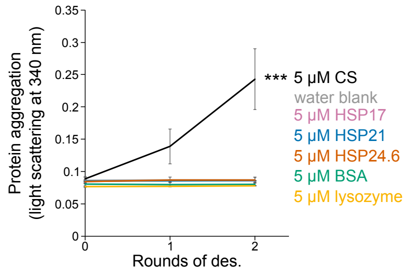

Figure S7. Only citrate synthase demonstrates desiccation-induced aggregation. 5 μM citrate synthase aggregates with successive rounds of desiccation ($p<0.001$, 1-way ANOVA, n=9). HSP17, HSP21, HSP24.6, BSA, and lysozyme (5 μM) each did not display appreciable aggregation following desiccation, suggesting they do not contribute to increases in A_{340} when in combination with CS. *** $p<0.001$.

Figure S8

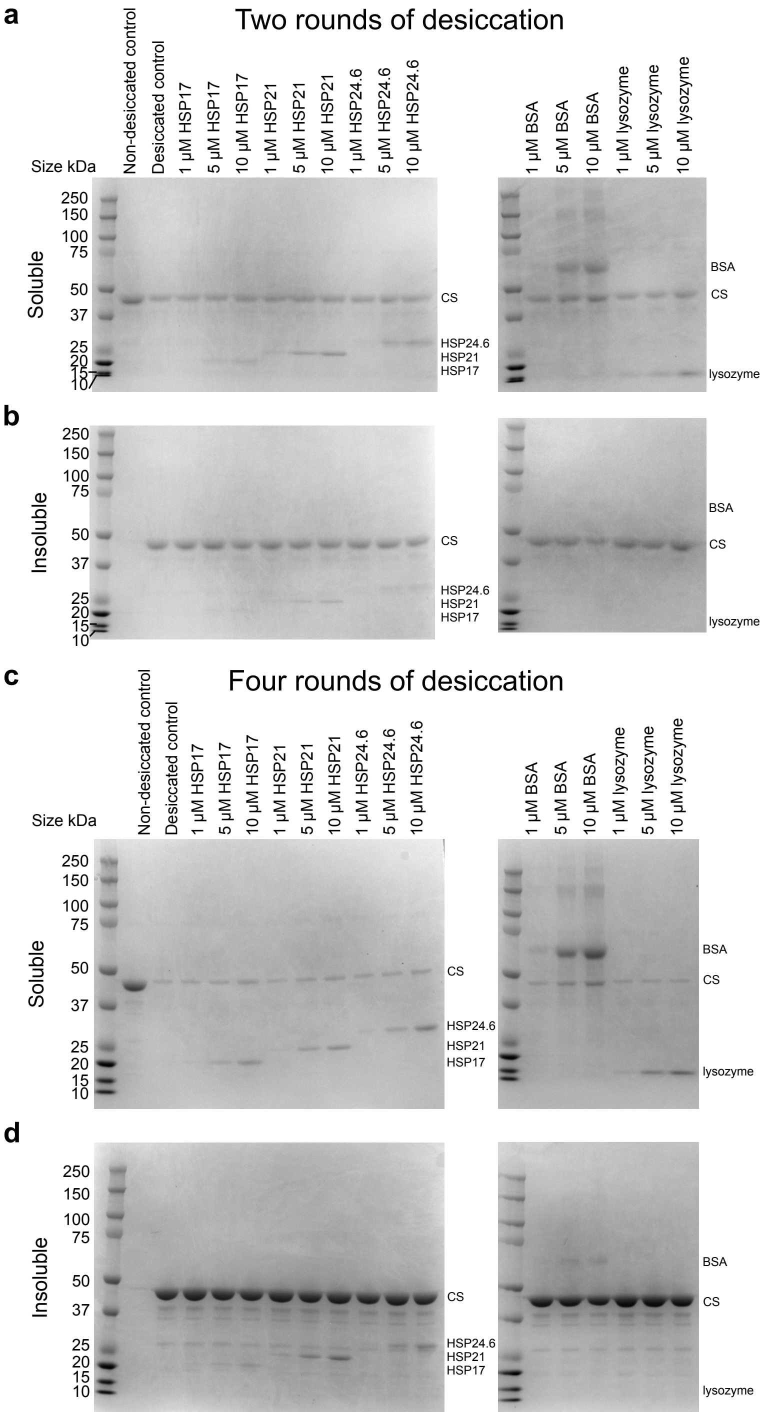

Figure S8. Solubility of citrate synthase and protein supplements after two and four rounds of desiccation. a) Soluble protein after two rounds of desiccation. b) Insoluble protein after two rounds of desiccation. c) Soluble protein after four rounds of desiccation. d) Insoluble protein after four rounds of desiccation.

Figure S9

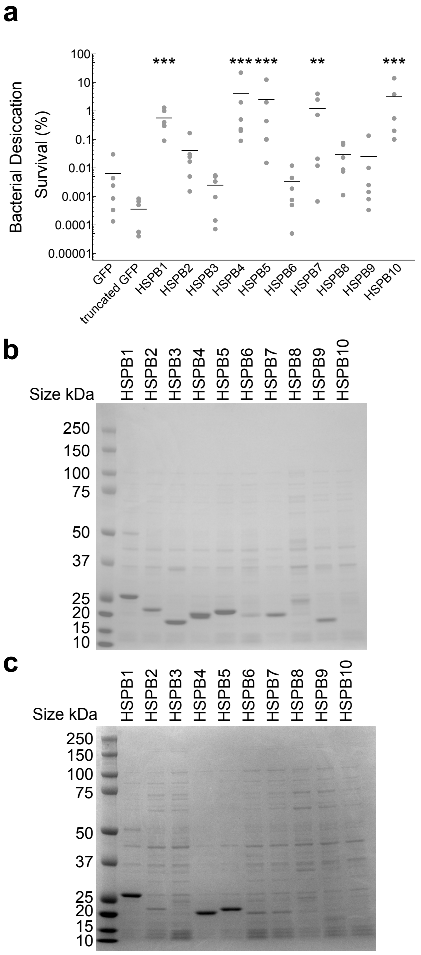

Figure S9. Some human sHSPs can improve bacterial desiccation tolerance. a) There was a significant difference in bacterial desiccation survival across strains expressing human sHSPs ($p<0.001$, 1-way ANOVA, $n=6$). Heterologous expression of HSPB1 ($p<0.001$), HSPB4 ($p<0.001$), HSPB5 ($p<0.001$), HSPB7 ($p=0.006$), and HSPB10 ($p<0.001$) in BL21 *E. coli* improved desiccation survival (Dunnett's test compared to GFP-expressing controls, $n=6$ for each). Total (b) and soluble (c) protein from bacterial lysates were assessed with SDS-PAGE. ** $p<0.01$, *** $p<0.001$.

Supplementary Table 1. sHSPs identified from the tardigrade *Hypsibius exemplaris*.

Protein	BV898 identifier	MW (kDa)	Length (aa)
HSP17	BV898_14399	17.43	151
HSP19	BV898_14400	19.428	169
HSP20	BV898_14401	20.199	176
HSP21	BV898_04512	21.419	187
HSP23	BV898_16197	22.953	205
HSP24.6	BV898_07709	24.612	217
HSP25	BV898_10301	25.088	220
HSP25.1	BV898_03100	25.142	219
HSP38	BV898_06733	38.113	351

Supplementary Table 2. Protein sequences of *H. exemplaris* sHSPs.

>He_HSP17
MRHMMRELNRMESQYSGQLDNTIQSHGPLTSLMDRSAIIPRIVDQNGQKIAQFNFNIQGFKPE
DVHIKTTDGRLVVSAKHEDKSEDHHAIREFRRMVTLPEGMQIEGMKSRLDPNGVLSIYAPYTPP
AIEHKQNLELPIHHERDPKALKEK

>He_HSP19
MFNRQVDPFGLGDILGDYDRQMRHMMRELNRMESQYSGQLDNTIQSHGPLTSLMDRSAIIPRI
VDQNGQKIAQFNFDIKGFKPQDVHIKTTDGRLVVSAKHEDKGEDHHAIREFRRMVTLPEGMQIE
GMKSRLDPNGVLSVYAPYTPPAIEHKQNELPIHHERDAKALK

>He_HSP20
MSLAPIGMFNRQVDAFGLGDILGDYDRQMRHMMRELNRMESQYEGQLDNTIQSQWPLTSLM
DRSAITPRILDQNGQKIAQFNFDIKGFKPQDVHIKTADGRLVVGAKHEDEGEDHHAIREFRRMV
TLPEGMQIEGMKSRLHPNGVLSVYAPYTPPAIEHKQNELPIHHERDAKALK

>He_HSP21
MSLLRWGPSYGSLWGRDIDPFFGLSDILGDYDRQMRQLTREFSRLENQFMGGQEEFPLGLTS
MVDRSAITPRIVDENGKKVAQYNFDIKGFRPEDVNIKTDQNGKLVVSARHEESDENHRAVREFY
RMVPLPEGVQLGDMKSHLRGDGVLTISAPLSLPALEQQQQQLKEIPIEHHGHNSINEKEKK

>He_HSP23
MALLPFFRRDQTDLPPDWDLSPWRNLDKTIGDFSSAPGFPAHFRSHVGGILRDMDNSIARMDE
EMRRVMMAAPGGAYNGPPGSLLSDLTHNISPSISTSSNGQQIAHYDFDVKGFRPEEITVKTQD
NLLEVIARHEERSPGREISREFKRTFTIPEGISPDELQGKLVQDGILRVEAPYRPPSALTSSSSEG
SYSYHPIPITHQY

>He_HSP24.6
MSRALARMIPQFFRNDLVEGPLRRGAGWGLSPFAGRPSSLGGMDFFDREINRMENLMAHMQ
NEMRRDLNSFLPLVRNQDYFDGGQNLINIVGENGKQKLQVKFQAENCKPEDIEVKTKGNLLEIR
TKQEDNGKDYSSYHEYTQMLTLPEGVNAEELTCKFEDGVVTLEAPYTKPALQAGETKDVPVEH
QPAEPVRKEIPIKREPASAADQVDQSRNK

>He_HSP25
MPAKNSRTYDTGPRRTTGDFYDLHDFRSFDELRKRLAKDVDQMEAELGRWRTPAWPTDQDA
SESYFIKHSLSHKTRSTSSSTTVYEDGRDSSESGDSDLGMSETMSPVPPKGILKRRSTPSPMG
TTRITEYRHSPKPKDTRVMDKERRPKDKLIFKFDVGDLRGADLAVQIMDTKLHVFAMDRKSDFR
REVQLPRTADPDSALSTLSNDGILRVEVILLS

>He_HSP25.1
MERMEESRSYSNLSHTSEVKVPVLQRETSVVEKEFGSIRERFDEEMRKMEDEMNRLRSDILEG
NKRLESSFKESRSTTTRTTSSKSNFGDGMGGLEPGGEYRTELKQWMDNLDSPLVSNHQDISG
HDGKCLRLRFDVSEYRPEEISVKTVDSKLKVHAKHEEKGEGKSVYREYNREFLLPKGTDPELIK
STLSKDGILTVEAPLPAIEPSEHRIPISNF

>He_HSP38
MSTTGNQNNVVEVRIPIMQRQTSVLDQDYGGSGGQFADEMRKMEEEMSRLTGQINEGNKRIA
SRIVQKTTTTTTRTTSGGGGGPVQQQPQPQPIQYSSPVQAGGNVQSQSSHTSTYSHQTTGTG
AQGQPQQQPSAVQWASPTPSGGLQQSSQQQHSTYTHSNSGHSPSPGPASVTGSNIQQHSS
HTSTSSHQTVTHHSNSGNQFDQFGQQQNQQQQFGGFNQQQNLDASNSQELQQWMGQLNS
PLIHNHQDIANQDGKCLRLRFDVSQYLPDEISVKTVDGKLRVHAKHEEKSEHKSAYREFNKEFS
LPIGTNPEAIKSTLSKDGILTVEAPLPGQQGQLGYGGQPALSNF
