# Supplementary material for: Tardigrade small heat shock proteins can limit desiccation-induced protein aggregation
Source: Commun Biol. 2023 Jan 30;6:121. doi: 10.1038/s42003-023-04512-y (PMC9887055; doi:10.1038/s42003-023-04512-y)
Supplement: Supplementary file 5 — Reporting Summary [file 42003_2023_4512_MOESM5_ESM.pdf]

nature portfolio

Corresponding author(s): Jonathan D. Hibshman

Last updated by author(s): Aug 4, 2022

Reporting Summary

Nature Portfolio wishes to improve the reproducibility of the work that we publish. This form provides structure for consistency and transparency in reporting. For further information on Nature Portfolio policies, see our Editorial Policies and the Editorial Policy Checklist.

Statistics

For all statistical analyses, confirm that the following items are present in the figure legend, table legend, main text, or Methods section.

n/a	Confirmed	
☐	☒	The exact sample size (*n*) for each experimental group/condition, given as a discrete number and unit of measurement
☐	☒	A statement on whether measurements were taken from distinct samples or whether the same sample was measured repeatedly
☐	☒	The statistical test(s) used AND whether they are one- or two-sided<br>*Only common tests should be described solely by name; describe more complex techniques in the Methods section.*
☒	☐	A description of all covariates tested
☐	☒	A description of any assumptions or corrections, such as tests of normality and adjustment for multiple comparisons
☐	☒	A full description of the statistical parameters including central tendency (e.g. means) or other basic estimates (e.g. regression coefficient) AND variation (e.g. standard deviation) or associated estimates of uncertainty (e.g. confidence intervals)
☐	☒	For null hypothesis testing, the test statistic (e.g. *F*, *t*, *r*) with confidence intervals, effect sizes, degrees of freedom and *P* value noted<br>*Give P values as exact values whenever suitable.*
☒	☐	For Bayesian analysis, information on the choice of priors and Markov chain Monte Carlo settings
☒	☐	For hierarchical and complex designs, identification of the appropriate level for tests and full reporting of outcomes
☒	☐	Estimates of effect sizes (e.g. Cohen's *d*, Pearson's *r*), indicating how they were calculated

Our web collection on statistics for biologists contains articles on many of the points above.

Software and code

Policy information about availability of computer code

Data collection	Data collection from the plate reader (BioTek) utilized Gen5 Microplate Reader and Imager Software version 3.08.
Data analysis	Analysis of data from previous RNA-seq experiments used publicly available software packages including Bowtie2, Featurecounts, and EdgeR. Other data analysis and visualization was conducted in Microsoft Excel and in Rstudio (ggplot2, Rmisc, DescTools).

For manuscripts utilizing custom algorithms or software that are central to the research but not yet described in published literature, software must be made available to editors and reviewers. We strongly encourage code deposition in a community repository (e.g. GitHub). See the Nature Portfolio guidelines for submitting code & software for further information.

Data

Policy information about availability of data

All manuscripts must include a data availability statement. This statement should provide the following information, where applicable:

- Accession codes, unique identifiers, or web links for publicly available datasets
- A description of any restrictions on data availability
- For clinical datasets or third party data, please ensure that the statement adheres to our policy

All data described in the manuscript are contained within the manuscript and supporting information. Raw data from previous RNA-seq experiments are available from the SRA database (SRP098585: GSM2472501 through GSM2472506 and PRJNA: SRX2663153, SRX2663154, SRX2527798, SRX2661843, SRX2661844, SRX2527616).

Human research participants

Policy information about studies involving human research participants and Sex and Gender in Research.

Reporting on sex and gender	N/A
Population characteristics	N/A
Recruitment	N/A
Ethics oversight	N/A

Note that full information on the approval of the study protocol must also be provided in the manuscript.

Field-specific reporting

Please select the one below that is the best fit for your research. If you are not sure, read the appropriate sections before making your selection.

☒ Life sciences ☐ Behavioural & social sciences ☐ Ecological, evolutionary & environmental sciences

For a reference copy of the document with all sections, see nature.com/documents/nr-reporting-summary-flat.pdf

Life sciences study design

All studies must disclose on these points even when the disclosure is negative.

Sample size	A minimum of three independent biological replicates were conducted for each experiment. We did not specifically conduct a power analysis.
Data exclusions	No data were excluded.
Replication	Replicates for experiments were conducted independently and are reported together along with the mean in most cases. For data, like TEM, for which individual images are presented we visualized proteins at different concentrations, in different buffers, and with different preparations to ensure reproducibility. Similarly, for mass photometry, we conducted experiments on different days and with different concentrations of protein and saw similar results to those presented.
Randomization	Samples were not randomized. Samples for individual replicates were set up in parallel.
Blinding	Blinding was not implemented in this study as the same investigator conducted and scored all experiments. Outputs from experiments were quantitative and did not require any scoring with subjective criteria.

Reporting for specific materials, systems and methods

We require information from authors about some types of materials, experimental systems and methods used in many studies. Here, indicate whether each material, system or method listed is relevant to your study. If you are not sure if a list item applies to your research, read the appropriate section before selecting a response.

Materials & experimental systems

n/a	Involved in the study	
☒	☐	Antibodies
☒	☐	Eukaryotic cell lines
☒	☐	Palaeontology and archaeology
☐	☒	Animals and other organisms
☒	☐	Clinical data
☒	☐	Dual use research of concern

Methods

n/a	Involved in the study	
☒	☐	ChIP-seq
☒	☐	Flow cytometry
☒	☐	MRI-based neuroimaging

Animals and other research organisms

Policy information about studies involving animals; ARRIVE guidelines recommended for reporting animal research, and Sex and Gender in Research

Laboratory animals	Hypsibius exemplaris (Z151)
Wild animals	N/A
Reporting on sex	N/A
Field-collected samples	N/A
Ethics oversight	N/A

Note that full information on the approval of the study protocol must also be provided in the manuscript.
